# Valproic acid-labeled chitosan nanoparticles promote recovery of neuronal injury after spinal cord injury

**DOI:** 10.18632/aging.103125

**Published:** 2020-05-28

**Authors:** Dimin Wang, Kai Wang, Zhenlei Liu, Zonglin Wang, Hao Wu

**Affiliations:** 1 Department of Neurosurgery, Xuanwu Hospital of Capital Medical University, Beijing, China; 2 School of Medicine, Zhejiang University, Hangzhou, China; 3 College of Basic Medical Sciences, Second Military Medical University, Shanghai, China

**Keywords:** spinal cord injury, chitosan nanoparticles, valproic acid, NF160, microglia

## Abstract

Chitosan nanoparticles have been recognized as a new type of biomaterials for treatment of spinal cord injury (SCI). To develop a novel treatment method targeted delivery injured spinal cord, valproic acid labeled chitosan nanoparticles (VA-CN) were constructed and evaluated in the treatment of SCI. Our results demonstrated that administration of VA-CN significantly promoted the recovery of the function and tissue repair after SCI. Moreover, we found treatment of VA-CN inhibited the reactive astrocytes after SCI. Furthermore, administration of VA-CN enhanced immunoreactions of neuronal related marker NF160, which suggested that VA-CN could promote the neuroprotective function in rats of SCI. The production of IL-1β, IL-6 and TNF-α were significantly decreased following treatment of VA-CN. Meanwhile, administration of VA-CN effectively improved the blood spinal cord barrier (BSCB) disruption after SCI. Administration of VA-CN could enhance the recovery of neuronal injury, suppress the reactive astrocytes and inflammation, and improve the blood spinal cord barrier disruption after SCI in rats. These results provided a novel and promising therapeutic manner for SCI.

## INTRODUCTION

Spinal cord injury (SCI) is a severe injury to the spinal cord that causes a loss of sensation, neurological function, autonomic function and muscle function in the body. Nearly 80 cases per million people suffer from spinal cord injury each year worldwide [1–3]. SCI consists mainly of the primary damage and the secondary damage. The primary injury includes the cell death, biochemical cascades, and tissue damage, which is usually caused by traffic accidents, violence and sports injuries. Furthermore, the secondary damage mainly contains the ischemic, inflammation, swelling and neural signal disorder, which is mediated by multiple neurodegenerative processes that accelerate the primary damage [4–6]. SCI involves in a series of pathophysiological processes such as metabolic disorder of extracellular matrix, reactive hyperplasia of glial cells and overexpression of inflammatory factors [7–9]. Among them, the reactive hyperplasia of glial cells is the main process of forming glial scars, which plays an important role in the development of SCI [10]. Glial fibrillary acidic protein (GFAP) is an intermediate filament protein that is mainly expressed in the cells from the central nervous system including astrocytes [11]. GFAP plays an essential role in maintaining the mechanical strength and the shape of astrocytes, which is recognized as a marker of reactive astrocytes [12]. Previous studies demonstrated that the expression of GFAP was increased after SCI in rats [13]. Although latest studies have revealed various effective manners and drugs in the treatment of SCI, the efficient carriers of transportation to achieve the specific location of spinal cord injury remained to solve [14, 15]. At present, many scholars have proposed the usage of different biological materials as neuroprotective drugs for SCI treatment [16–18]. Methylprednisolone (MP) is the only clinical drug for SCI treatment, which is still controversy in efficacy and safety of treating SCI [19–22]. A recent study adopted MP-loaded poly lactic-co-glycolic acid (PLGA) nanoparticles in the injured spinal cord to reduce the inflammation and improve damage level after contusion SCI [23]. However, the usage of systemic high-dose of MP in the acute SCI has the risk of serious side effects including gastric bleeding, sepsis, pneumonia, and acute corticosteroid myopathy and wound infections, with just modest improvements in neurological recovery [24–26]. Thus, the newly effective therapeutic method is urgent to investigate in the treatment of SCI.

Synthetic nano-sized polymers have been recently recognized as a new type of neuroprotective agents for treatment of early SCI [27, 28]. Valproic and chitosan nanoparticles were separately reported to effectively improve the recovery of the function and tissue repair after SCI as the intervention factors [29, 30]. Previous studies demonstrated that chitosan nanoparticles and its modifications promoted functional restoration of traumatically injured spinal cord after SCI [31, 32]. On the other hand, valproic acid was showed microglia neuroprotection and involved in rat cauda equina injury [33]. The latest report demonstrated that valproic acid alleviated the inflammation induced by traumatic spinal cord injury via STAT1 and NF-κB dependent of HDAC3 signaling pathway [34]. Therefore, the neuroprotective effect of valproic acid combined chitosan nanoparticles and their fundamental mechanism on the nervous system after SCI need further investigate. Here, we found valproic acid labeled chitosan nanoparticles treatment promoted the recovery of the function and tissue repair and inhibited the reactive astrocytes after SCI. Meanwhile, valproic acid labeled chitosan nanoparticles treatment enhanced the blood spinal cord barrier integrity after SCI. The results provided a new potential therapeutic approach for the clinical treatment of SCI.

## RESULTS

### Characteristics of valproic acid labeled chitosan nanoparticles

**Figure 1:**
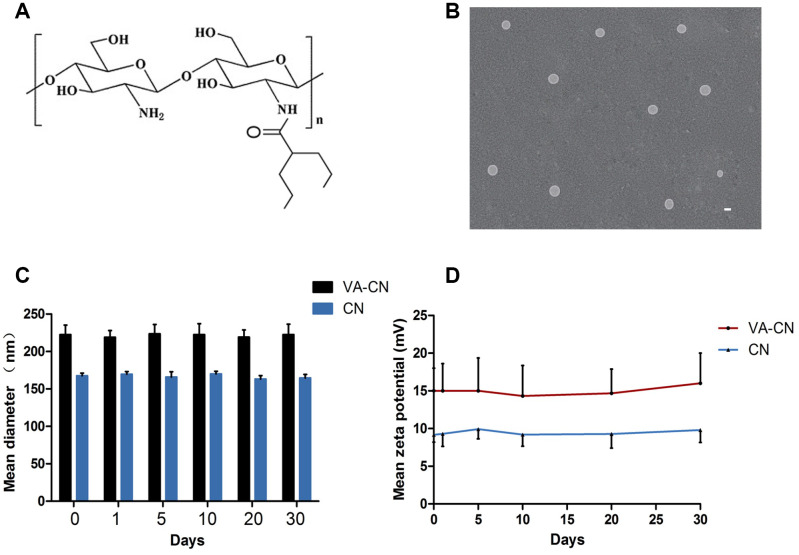
**Valproic acid modified chitosan nanoparticles (VA-CN).** (**A**) Chemical structure of VA-CN nanoparticles. (**B**) TEM image of VA-CN nanoparticles (Scale bar: 200 nm). (**C**) Sizes of VA-CN and CN nanoparticles were observed for different time points during one mouth. (**D**) Zeta potential of VA-CN and CN nanoparticles were detected by ZetaPlus for different time points during one mouth.

As shown in Figure 1A, valproic acid was incorporated to chitosan nanoparticles through coupling carboxyl to amino group (Figure 1A). The morphology of valproic acid labeled chitosan nanoparticles was observed by transmission electron microscopy. The result revealed the spherical shape of valproic acid labeled chitosan nanoparticles were sized at 200 nm or so (Figure 1B). To determine the stability and surface charge of valproic acid labeled chitosan nanoparticles, the particles were incubated at 4°C for 30 days. The sizes of valproic acid labeled chitosan nanoparticles were around 220 nm and the zeta potential of valproic acid labeled chitosan nanoparticles was found to be nearly 15 mV, which suggested that the stability of the particles was successfully maintained at low temperature for one month (Figure 1C and 1D). In addition, the sizes of chitosan nanoparticles were around 170 nm and their zeta potential were nearly 10 mV at 4°C for 30 days (Figure 1C and 1D).

### VA-CN targeted delivery to injured spinal cord

**Figure 2:**
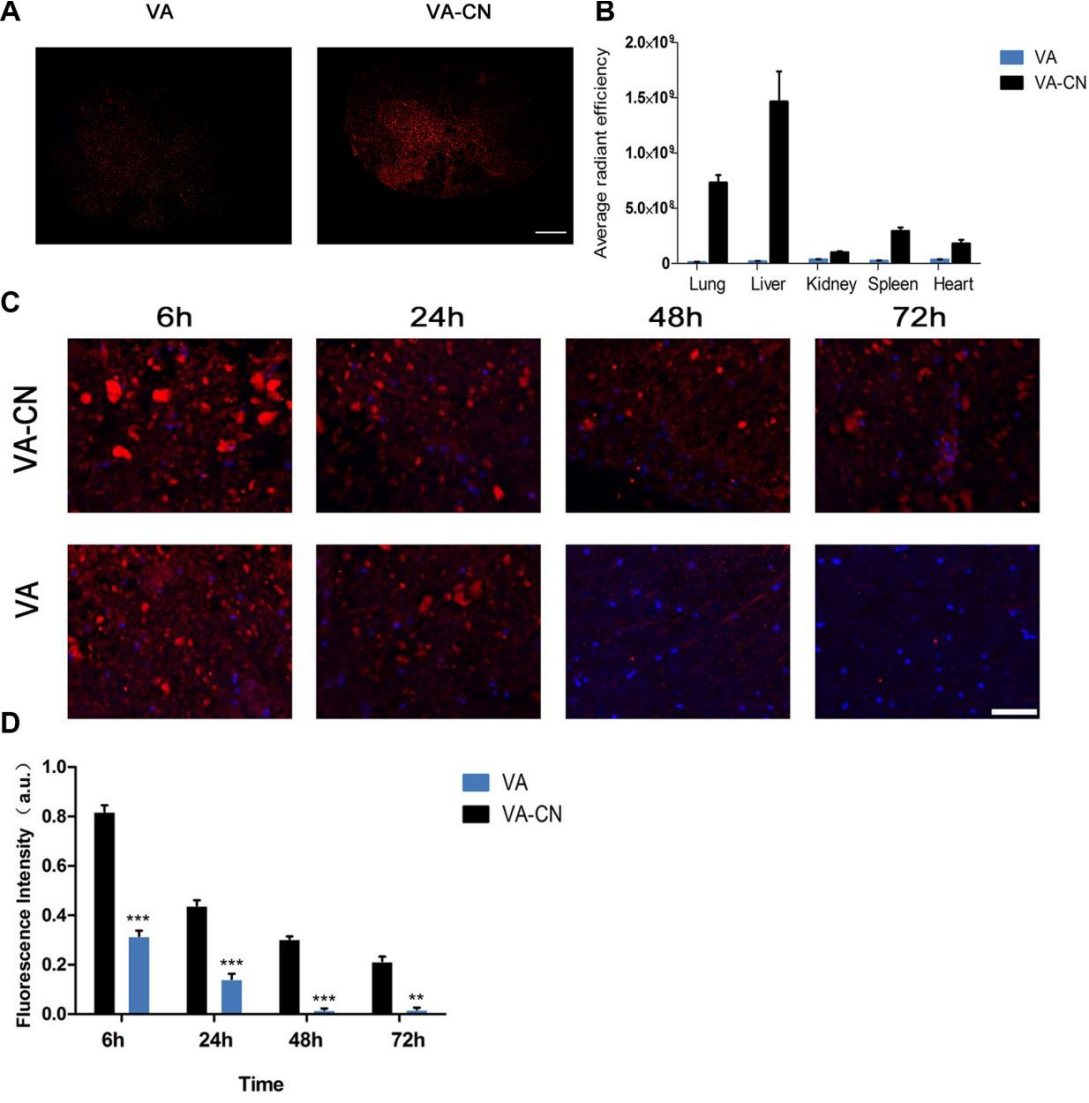
**VA-CN targeted delivery to injured spinal cord.** (**A**) Fluorescence images of VA-CN-Cy5.5 and VA-Cy5.5 in injured spinal cord at 24h after SCI (Scale bar: 500 μm) n=8 per group. (**B**) Quantification of VA-CN-Cy5.5 and VA-Cy5.5 in organ distribution, n=4 per group. (**C**) Fluorescence images of VA-CN-Cy5.5 and VA-Cy5.5 in injured spinal cord, (Scale bar: 100 μm). (**D**) Quantitative results of fluorescence intensity of Cy5.5. n=8 per group, ** p<0.01 VS VA group, *** p<0.001 VS VA group.

To investigate the effect of VA-CN on targeted delivery to injured spinal cord, the Cy5.5 was labeled to VA-CN polymer at room temperature. The Cy5.5 labeled VA-CN and VA were treated the rats of SCI by intravenous administration and quantified in the injured spinal cord and different organs at 24 h after SCI (Figure 2A and 2B). The concentration of the particles was measured at injured spinal cord for various time points by detecting the fluorescence intensity of Cy5.5. The result demonstrated that the fluorescence intensity was gradually decreased in the two groups with the increased treatment time (Figure 2C). The effectiveness and maintenance of delivery to injured spinal cord were significantly enhanced by the treatment of VA-CN compared with VA treatment group, which estimated through the fluorescence intensity of Cy5.5 at injured spinal cord (Figure 2D). The fluorescence intensity was seldom detected in the VA group after 48 h post treatment (Figure 2C). Moreover, the distribution of VA-CN was testified in the spinal cord of uninjured rats and the fluorescence intensity of Cy5.5 was obviously detected in the treatment of VA-CN-Cy5.5 for 48 h, but not in the treatment of VA-Cy5.5 (Supplementary Figure 1). In addition, H&E staining result showed no morphological difference between the Sham rats and the VA-CN treated SCI rats (Supplementary Figure 2), which suggested VA-CN revealed no adverse effects in various organs of the rats.

### VA-CN enhanced the function and tissue recovery after SCI

**Figure 3:**
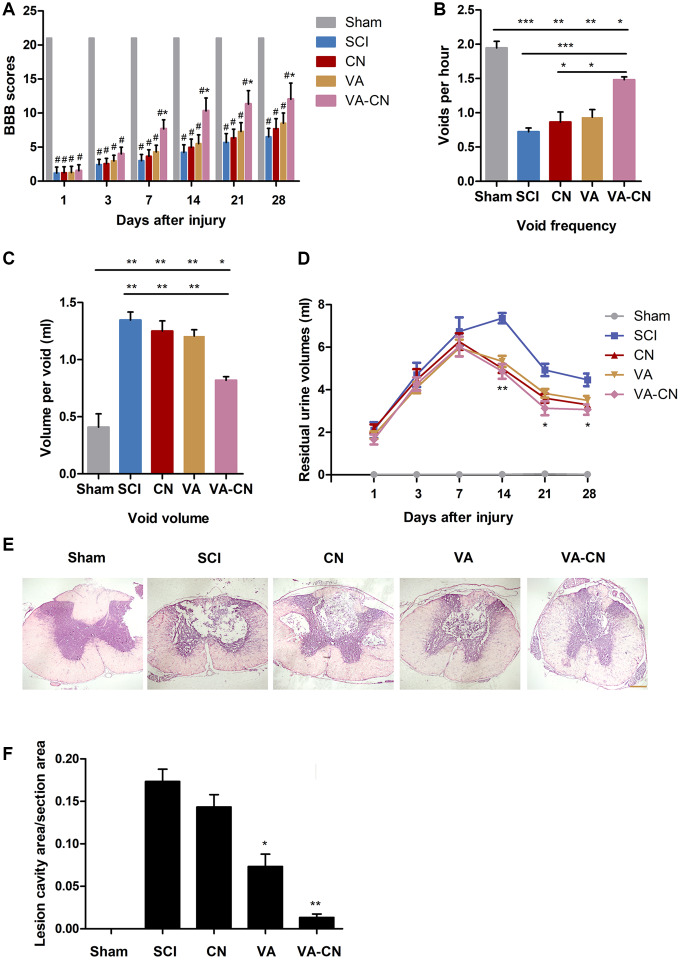
**VA-CN administration promoted recovery after SCI.** (**A**) Basso, Beattie and Bresnahan (BBB) scores were evaluated at different time points after injury in Sham rats (n=9), SCI rats (n=10), CN treated rats (n=10), VA treated rats (n=12), and VA-CN treated rats (n=11). Six rats with perineal infections, limb wounds, or tail and foot grazing were eliminated from the test. * p<0.05 VS SCI group, # p<0.001 VS Sham group. (**B**, **C**) Void frequency and average void volume were tested at 4 weeks after SCI. n=6 per group, * p<0.05, ** p<0.01, *** p<0.001. (**D**) Residual urine volumes were recorded at different time points after injury. n=10 for Sham group, n=12 per experiment group, * p<0.05, ** p<0.01 VS SCI group. (**E**) The HE staining was performed at 4 weeks after injury (Scale bar: 100 μm). (**F**) The lesion cavity area was quantified in the injured spinal cords. n=10 for Sham group, n=12 per experiment group, * p<0.05, ** p<0.01 VS SCI group.

To investigate the effect of VA-CN on SCI, we assessed the tissue and function repair by treatment of VA-CN after SCI. The BBB scores of all experimental groups decreased significantly compared with the sham group (Figure 3A). After treatment of VA-CN for one week, the BBB scores were significantly increased compared with the SCI group (Figure 3A). On the other hand, VA or CN alone treatment resulted in no significant increase of the BBB scores for different time points compared with the SCI group (Figure 3A). Moreover, VA-CN treatment remarkably enhanced void frequency and decreased void volume compared with the control group at 4 weeks after SCI, which suggested the improved connections between the control system of brain and the bladder (Figure 3B and 3C). VA treatment just slightly improved the connections compared with the SCI group (Figure 3B and 3C). Furthermore, the residual urine volumes were also measured at different time points and the result revealed that VA-CN treatment led to a significant decrease in residual urine volumes after two weeks post injury compared with the SCI group (Figure 3D). The residual urine volumes were gradually decreased after one week post injury in the VA treatment and two weeks post injury in the SCI group, and VA or CN alone treatment showed no significant change in residual urine volumes for different time points compared with the SCI group (Figure 3D). In order to explore the effect of VA-CN on tissue recovery after SCI, the H&E staining was performed at 4 weeks after injury. The result demonstrated that administration of VA-CN significantly reduced the lesion cavity volume, and VA treatment slightly improved the lesion cavity volume compared the SCI group (Figure 3E and 3F). In addition, the dispersed structure and hemorrhage were apparently improved by the VA-CN treatment when compared with the SCI, VA, and CN treatment group (Figure 3E).

### VA-CN reduced astrocytic reactivity after SCI

**Figure 4:**
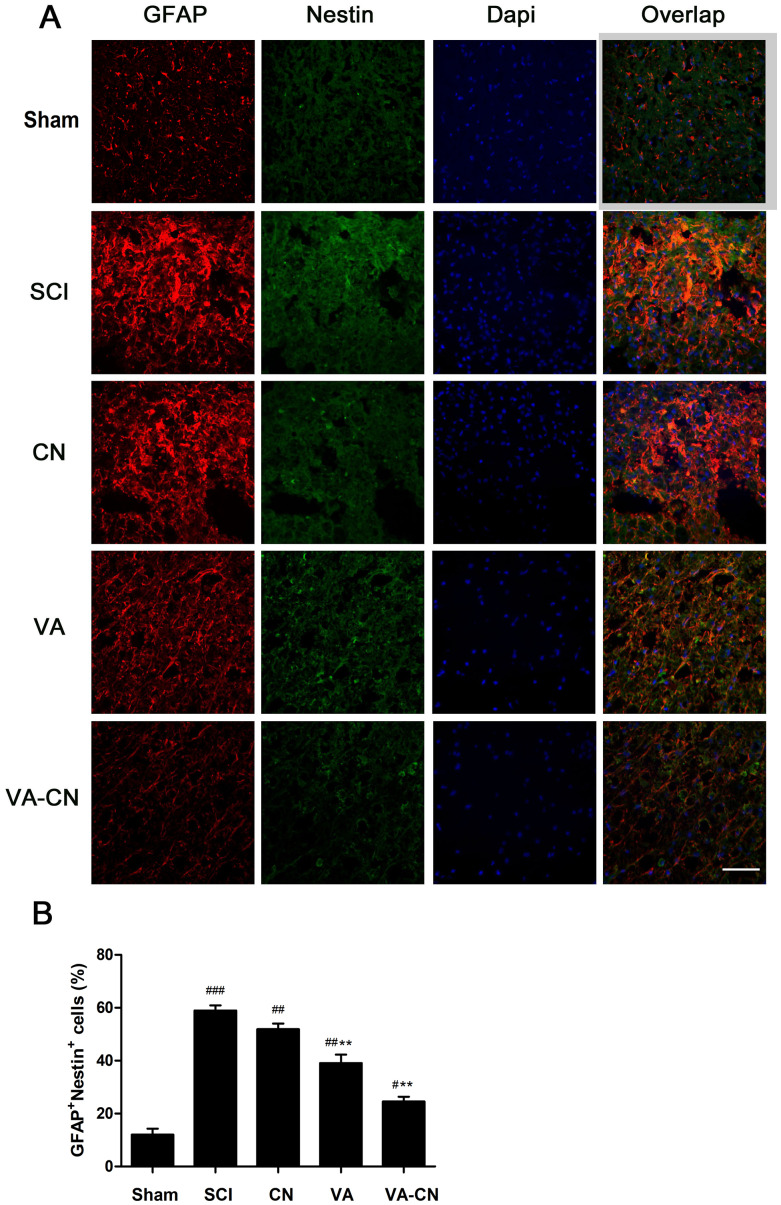
**VA-CN reduced astrocytic reactivity in the injuried spinal cord grey matter.** Levels of astrocytic reactivity was estimated by double immunostaining for GFAP and nestin. (**A**) Confocal images of injury sites analyzed for overlap of GFAP (red), nestin (green) and Dapi (blue), Scale bar: 50 μm. (**B**) The astrocytic reactivity was quantified by GFAP^+^nestin^+^ cells, n=6 per group, * p<0.05, *** p<0.001 VS SCI group, ### p<0.001 VS Sham group, ## p<0.01 VS Sham group, # p<0.05 VS Sham group.

To investigate the effect of VA-CN on astrocytic reactivity, the astrocyte reactivity was measured following VA-CN treatment after SCI. In comparison to the Sham group, the GFAP^+^nestin^+^ cells were significantly enhanced in SCI rats (Figure 4A and 4B). Moreover, we found the immunoreaction of GFAP and Nestin was reduced obviously by the treatment of VA-CN, which suggested that VA-CN might inhibit the reactive astrocytes in rats of SCI (Figure 4A). Furthermore, the result revealed that GFAP^+^nestin^+^ cells were significantly decreased following treatment with VA-CN compared with SCI group (Figure 4A and 4B). Interestingly, VA alone treatment also lead to a slight decrease in percentage of GFAP^+^nestin^+^ cells, which were not significant changes in the CN treatment group when compared with the SCI rats (Figure 4A and 4B). These results demonstrated that VA-CN administration significantly reduced the levels of astrocyte reactivity compared to the SCI rats.

### VA-CN promoted neuroprotection and inhibited inflammation after SCI

**Figure 5:**
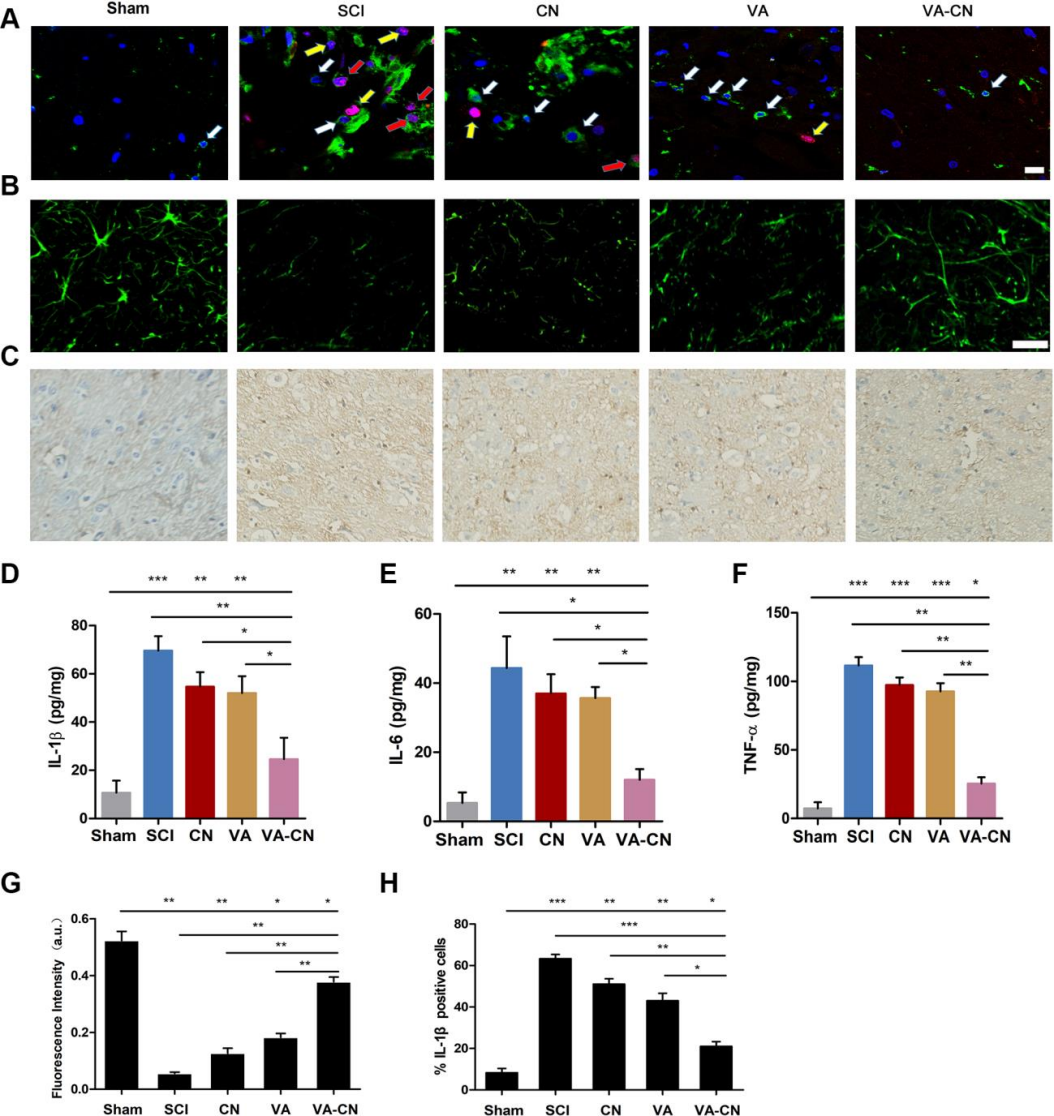
**VA-CN promoted neuroprotection after SCI.** (**A**) Co-labeled CD11b (green) immunoreactive, Ki67 marker (red) and Dapi (blue) in the spinal cord of rats. White arrows represent microglia, yellow arrows represent proliferated cells and red arrows represent the proliferation of microglia cells (Scale bar: 20 μm), n=6 per group. (**B**) Florescence images of NF160 in injured spinal cord at 28 day after SCI (Scale bar: 50 μm). (**C**, **H**) Representative images for IL-1β immunohistry (200× magnification) at 7 days after injury and the IL-1β positive cells were quantified. n=6 per group, * p<0.05, ** p<0.01, *** p<0.001. (**D**–**F**) Quantification of IL-1β, IL-6 and TNF-α production was evaluated at 7 days after injury, n=6 per group, * p<0.05, ** p<0.01, *** p<0.001. (**G**) Intensify quantification of NF160 florescence in injured spinal cord at 28 day after SCI, n=6 per group, * p<0.05, ** p<0.01.

To investigate the effect of VA-CN on the proliferation of microglia after SCI, the injured spinal cord was co-labeled with CD11b and Ki67. The result revealed that VA-CN treatment lead to a decrease in the number of microglia and the proliferation of microglia (Figure 5A). To further estimate the effect of VA-CN on the nerve after SCI, the neuronal related marker NF160 was detected by histological analysis. The result indicated that VA-CN treatment enhanced the immunoreaction of NF160, while VA administration revealed slight increase in the NF160 immunoreaction compared with the SCI group (Figure 5B and 5G). The immunohistry analysis revealed that VA-CN significantly decreased the expression of IL-1β compared with SCI group, whereas CN and VA treatment also reduced the IL-1β positive cells in the injured spinal cord after SCI (Figure 5C and 5H). Furthermore, the inflammation induced by SCI was assessed by the production of IL-1β, IL-6 and TNF-α at 7 days after injury. The result revealed that VA-CN significantly decreased the secretion of IL-1β, IL-6 and TNF-α compared with the SCI, CN and VA treatment group, whereas VA administration effectively reduced the production of IL-1β and revealed no significant difference in the IL-6 and TNF-α secretion at 7 days after injury (Figure 5D–5F).

### VA-CN enhanced the integrity of blood spinal cord barrier after SCI

**Figure 6:**
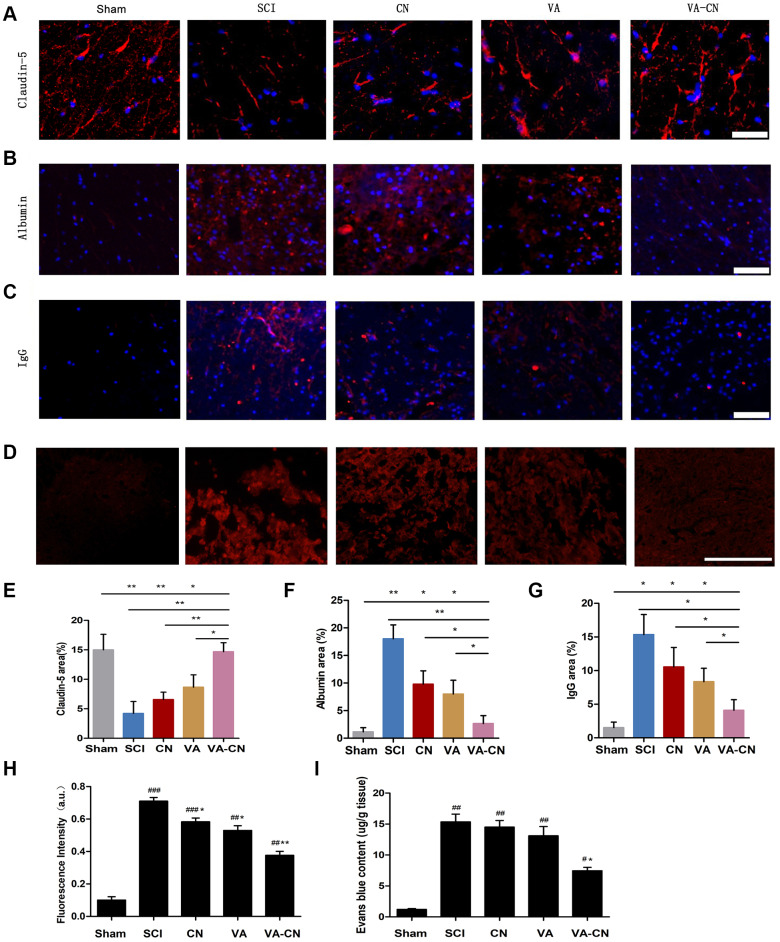
**VA-CN enhanced the integrity of blood spinal cord barrier after SCI.** The blood spinal cord barrier integrity was measured by Claudin-5, Albumin, IgG expression at 4 weeks (n=6 per group) and Evans blue extravasation at 24 h (n=4 per group) after injury. (**A**, **E**) Claudin-5 immunoreactivity and quantification to the spinal cord of rats (Scale bar: 100 μm), * p<0.05, ** p<0.01. (**B**, **F**) Albumin immunoreactivity and quantification to the spinal cord of rats (Scale bar: 100 μm), * p<0.05, ** p<0.01. (**C**, **G**) IgG immunoreactivity and quantification to the spinal cord of rats (Scale bar: 100 μm), * p<0.05. (**D**, **H**) Evans blue extravasation and quantification in the spinal cord of rats (Scale bar: 50 μm), * p<0.05, ** p<0.01 VS SCI, ## p<0.01 VS Sham, ### p<0.001 VS Sham. (**I**) Quantification data of Evans blue content in the spinal cord (μg/g), * p<0.05 VS SCI, # p<0.05 VS Sham, ## p<0.01 VS Sham.

The BSCB restricts the access of erythrocytes and plasma components in the central nervous system, which is damaged after SCI. Thus, the repair of BSCB disruption is necessary to estimate in various treatments after SCI. The representative markers immunoreaction of BSCB integrity, including Claudin-5, Albumin and IgG, were detected and the result revealed that VA-CN treatment led to a significant increase of Claudin-5 immunoreaction compared with the control, CH and VA group after SCI (Figure 6A and 6E). Moreover, the immunoreactive intensity of Albumin was significantly decreased in the treatment of VA-CN in comparison to the control, CH and VA group after SCI (Figure 6B and 6F). On the other hand, administration of VA-CN resulted in a decrease of IgG immunoreaction compared with the control, CH and VA group after SCI (Figure 6C and 6G). Moreover, to further evaluate the effect of VA-CN on the BSCB permeability, Evans blue extravasation was performed after SCI. The Evans blue fluorescence and content results showed that VA-CN significantly inhibited the extravasation of EB after SCI (Figure 6D, 6H and 6I). These results suggested that VA-CN treatment could enhance the integrity of blood spinal cord barrier after SCI.

## DISCUSSION

PLGA-MP labeled nanoparticles administration has been shown to significantly reduce lesion volume and improve recovery of SCI compared with the systemic MP delivery in rats [23]. A number of previous studies have demonstrated that the therapeutic effects of valproic acid delivery on SCI were valid through various mechanisms, including attenuated inflammation induced by SCI, mediated neuroprotection and neurogenesis, promoted neurite outgrowth by stimulating overexpression of microtubule-associated protein 2, reduced autophagy and enhanced motor function, attenuated blood-spinal cord barrier disruption by inhibiting matrix metalloprotease-9 activity [35–37]. However, the utilization of highly dose valproic acid delivery by intravenous administration was controversial since the risk of side effects and limited effectiveness in SCI [38, 39]. Synthetic nano-sized polymers have been shown to effective administration containing various types of drugs for treatment of SCI [40, 41]. Therefore, it is necessary to explore the therapeutic effects and detailed mechanisms of valproic acid combined with nanoparticles delivery on SCI in rats.

In this study, we developed a novel approach of chitosan nanoparticles carried valproic acid for the first time in the treatment of injured spinal cord in rats. Previous studies have demonstrated that potential advantages of chitosan nanoparticles administration containing various types of drugs revealed biocompatibility, availability and ease of functionalization compared with conventional systemic delivery [42]. A recent study has revealed that chitosan nanoparticles exerted neuroprotection by its membrane sealing effects in oxidative stress-mediated injury [43]. Our results showed that administration of VA-CN significantly promoted the recovery of the function and tissue repair and inhibited the reactive astrocytes after SCI. On the other hand, previous studies have shown that VA potentiated neuroprotection and function recovery after SCI [30, 34]. However, our results revealed that VA alone treatment just slightly improved the injured area, neuronal injury, reactive astrocytes, inflammation, and blood spinal cord barrier disruption, which might be relevant to the low dose of VA and intravenous administration manner in this study. The effectiveness and maintenance of delivery to injured spinal cord were significantly enhanced by administration of VA-CN through evaluating the fluorescence intensity of Cy5.5 at injured spinal cord. Interestingly, the distribution of VA-CN was also revealed in the spinal cord of uninjured rats. *In vivo* toxicity analysis demonstrated VA-CN treatment resulted in no morphological changes in the liver, lung, spleen, kidney, and heart of SCI rats, which suggested that accumulation of VA-CN cause no damage in various organs of the rats. Moreover, the BBB scores, connections between the control system of brain and the bladder, lesion cavity volume were significantly improved by treatment of VA-CN after SCI. Furthermore, administration of VA-CN effectively increased the immunoreaction of neuronal related marker NF160 and remarkably reduced the reactive astrocytes in rats of SCI. The production of IL-1β, IL-6 and TNF-α were significantly decreased following treatment of VA-CN. In addition, administration of VA-CN also effectively improved the blood spinal cord barrier disruption after SCI through estimating the BSCB representative markers Claudin-5, Albumin and IgG expression and Evans blue extravasation. Our results indicated the promising potential of VA-CN nanoparticles for treating SCI in clinic. We presented evidence that administration of VA-CN exerted the potential to improve recovery of neuronal injury and motor function after SCI by intravenous route, which was relatively simple to implement and provided new insight into the benefits of administration of VA-CN and encouraged the clinical application of this treatment. However, further work is needed to validate the effectiveness by assessing preclinical outcomes.

## CONCLUSIONS

Taken together, effective delivery of VA-CN to the injured spinal cord decreased lesion cavity volume and improved function recovery compared with systemic VA delivery. Based on our results, administration of VA-CN could enhance the recovery of neuronal injury, suppress the reactive astrocytes and inflammation, and improve the blood spinal cord barrier disruption after SCI in rats. These results maximized the therapeutic effectiveness of VA in the treatment of SCI. Although further studies are needed to more precisely determine the exact therapeutic mechanism and to assess how dosage, administration frequency and timing of treatment with VA-CN may affect the clinical outcome, this study find a new perspective for the treatment of SCI.

## MATERIALS AND METHODS

### Preparation and characterization of valproic acid labeled chitosan nanoparticles

Conjugation of valproic acid and chitosan nanoparticles was shown in Figure 1A. The valproic acid and chitosan nanoparticles were conjugated by coupling carboxyl to amino group. Briefly, 10 mg valproic acid diluted by 5 ml dimethyl sulfoxide (DMSO) was added to 10 ml of 1 mg/ml chitosan solution in the presence of 1-ethyl-3-(3-dimethylaminopropyl)-carbodiimide hydrochloride (EDC) and N-hydroxysuccinimide (NHS) modification reagents for 24 h at room temperature. The resulting solutions were dialyzed for 48 h to isolate conjugates. The morphology of conjugates was analyzed by transmission electron microscopy (TEM). The surface charges of VC-CN nanoparticles in distilled water were determined using a Zetaplus analyzer (Brookhaven Instrument Co., CA).

### Cy5.5-labeled VC-CN nanoparticles

Cy5.5 was dissolved in DMSO and added to VC-CN or VC solution for 6 h at room temperature in the dark. The solution was performed with dialysis against distilled water. The amounts of Cy5.5 in the VC-CN and VC treatment in the injured spinal cord, uninjured spinal cord, and various organs were determined by fluorescence.

### Animals

Adult male rats (180 to 220 g, Sprague-Dawley, Harlan) were provided by the Animal Center of Capital Medical University. All of the animals were treated humanely and with regard for the alleviation of suffering. This study was carried out in accordance with the guidelines of the Care and Use of Laboratory Animals of the National Institutes of Health. All experimental protocols described in this study were approved by the Ethics Review Committee for Animal Experimentation of Capital Medical University.

### Animal model of SCI

The rats were anesthesia by 4 % isoflurane. A laminectomy was performed at the thoracic vertebra level 10 (T10) after shaving and cleaning until fully recovered from the anesthesia. Spinal cord contusion was induced using a weight-drop apparatus, where a guided 5g rod was dropped from a height of 80 mm onto the exposed cord, representing moderate SCI. After surgery, the muscles were sutured in layers and the skin incision was closed with silk threads. Penicillin G (40,000 U, i.m.) was administrated daily for 3 days to prevent infection. Rats that died for any reasons were excluded from the experiment, and a new one was added to the study. The sham rats were subjected to laminectomy without SCI.

### Experimental groups and interventions

Fifty-eight rats were randomly assigned to five groups: Sham rats (n=10), SCI rats (n=12), CN-treated SCI rats (n=12), VA-treated SCI rats (n=12) and VA-CN-treated SCI rats (n=12). 15 mg/kg concentration of VA-CN, 15 mg/kg concentration of CN and 80 mg/kg concentration of VA were intravenously administered daily for 5 days and started at 1 h after injury. After injury, the rats of SCI model group were injected with saline solution in the tail vein. The other groups were administrated with 15 mg/kg concentration of VA-CN, 15 mg/kg concentration of CN or 80 mg/kg concentration of VA (500 ul in saline) through a single intravenous tail vein injection. In addition, four Sham rats and four VA-CN-treated SCI rats were used to evaluate the side effect of VA-CN *in vivo*.

### Behavioral assessment

The locomotor activity was assessed at 1, 3, 7, 14 and 28 days post-injury using the Basso Beattie Bresnahan (BBB) locomotor score method. The final score for each animal was obtained by averaging values from both investigators. Rats with perineal infections, limb wounds, or tail and foot grazing were eliminated from the test.

### Urine collection

The residual urine volumes were detected from morning volumes. To obtain urine from SCI rats at various times, animals were anesthetized with 4% isoflurane and administered 2 ml PBS intravenously via the tail vein to facilitate urine production. After 1 hour, urine was collected via transurethral catheterization. The void frequency per hour and volume per void were collected using constant infusion of room temperature PBS through the catheter into the bladder at 4 weeks after SCI.

### Histopathological analysis

The 5 μm longitudinal sections were made from the paraffin embedded blocks and stained with hematoxylin solution for 5 min. Then the sections were stained with eosin solution for 3 min and followed by dehydration with graded alcohol and clearing in xylene. The mounted slides were then observed and photographed using a light microscope (Nikon, Tokyo, Japan). Images were collected at 100× magnification. The lesion cavity volume was evaluated using H&E staining under the light microscope. *In vivo* toxicity analysis, the liver, lung, spleen, kidney, and heart were embedded into paraffin. Sections of 5 m thickness were stained with haematoxylin and eosin to evaluate the *in vivo* toxicity of VA-CN (400× magnification).

### Tissue preparation and ELISA analysis

For the enzyme-linked immunosorbent assay, rats were sacrificed and the spinal cord was immediately dissected on ice. 10-mm-long spinal cord segments containing the injury epicenter were removed as quickly as possible. The samples were then flash-frozen and stored in liquid nitrogen. The samples were subjected to measure the cytokines production of IL-1β, IL-6 and TNF-α at 7 days post-injury by ELISA according to manufacturer’ s instructions (Cusabio Biotech Co, Wuhan, China). All assays were performed in duplicates using recommended buffers, diluents, and substrates.

### Immunocytochemistry

At 28 days post injury, the rats were anesthetized and transcardially exsanguinated with 150 ml physiological saline followed by fixation. A 1 cm spinal cord segment at the lesion center was dissected and then fixed 4 h by 4 % paraformaldehyde in PBS. The cord segments were embedded in tissue embedding medium, and 30 m sagittal sections were cut on a cryotome and mounted onto glass slides. Albumin (cat. #EPR20195) and IgG (cat. #ab150116) (Abcam, Cambridge, MA, USA), claudin-5 (cat. #sc-374221) antibodies (Santa Cruz Biotechnology, CA, USA) were used to evaluate BSCB integrity. CD11b (Cat. #NB110-89474) antibodies (Novus Biologicals, Littleton, CO, USA) and Ki67 (cat. #ab16667) antibodies (Abcam, Cambridge, MA, USA) were used to evaluate activated microglia. IL-1β (Cat. #ab9722) antibodies (Abcam, Cambridge, MA, USA) were used to evaluate inflammation. NF160 (cat. #ab7794), GFAP (cat. #ab4674), Nestin (Cat. #ab134017) antibodies (Abcam, Cambridge, MA, USA) were used to evaluate neuronal restore and astrocyte reactivity. Sections were incubated in a hydrogen peroxide solution (0.3%) for 1 hour at room temperature. Second antibodies were visualized using the fluorescence microscopy (Nikon, Tokyo, Japan) or visualized using confocal microscopy (Zeiss 710 and LSM software).

### Measurement of Evans blue extravasation

After SCI, Evans Blue dye (2% w/v in saline, Sigma-Aldrich) was injected intravenously under anesthesia. 1 h after the injection, rats were perfused with saline and rinsed thoroughly until no more blue dye flew out of the right atrium. The spinal cords were acquired and the Evans Blue content and Evans Blue fluorescence were used to measure Evans Blue extravasation. The spinal cord tissue was weighed and soaked in methanamide for 24 hours and then centrifuged. The absorption of the supernatant was measured at 620 nm with a microplate reader (Molecular Devices). The content of EB was measured as micrograms per gram of spinal cord tissue. The spinal cord tissue was fixed in 4% paraformaldehyde and kept frozen. Evans Blue staining was visualized using a light microscope (Nikon, Tokyo, Japan).

### Statistical analysis

Results are presented as the means ± S.D. from at least three independent experiments. The statistical differences were calculated by the Student’s *t*-test or one-way ANOVA analysis of variance with Dunnett’s test. * P<0.05 was considered significant.

## Supplementary Material

Supplementary Figures
