# Supplementary material for: Valproic acid-labeled chitosan nanoparticles promote recovery of neuronal injury after spinal cord injury
Source: Aging (Albany NY). 2020 May 28;12(10):8953–67. doi: 10.18632/aging.103125 (PMC7288920; doi:10.18632/aging.103125)
Supplement: Supplementary Figures [file aging-12-103125-s001..pdf]

SUPPLEMENTARY FIGURES

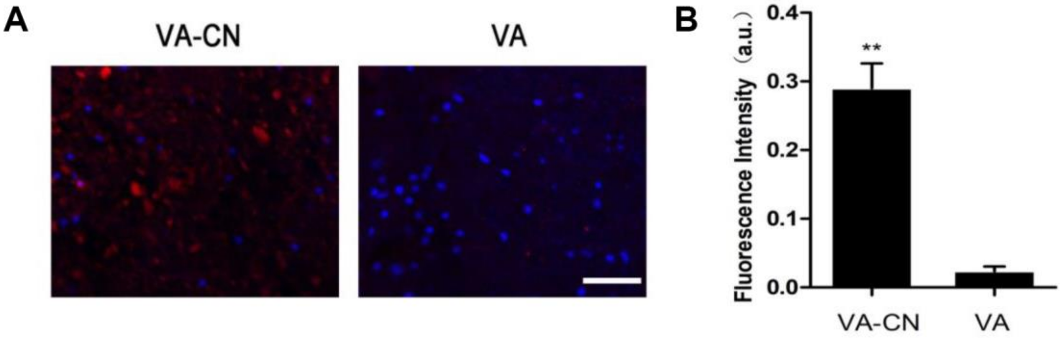

Supplementary Figure 1. The distribution of VA-CN in the spinal cord of uninjured rats. (**A**) Fluorescence images of VA-CN-Cy5.5 and VA-Cy5.5 in uninjured spinal cord at 48 h after treatment (Scale bar: 100 µm), n=4 per group. (**B**) Quantitative results of fluorescence intensity of Cy5.5, n=4 per group. ** p<0.01.

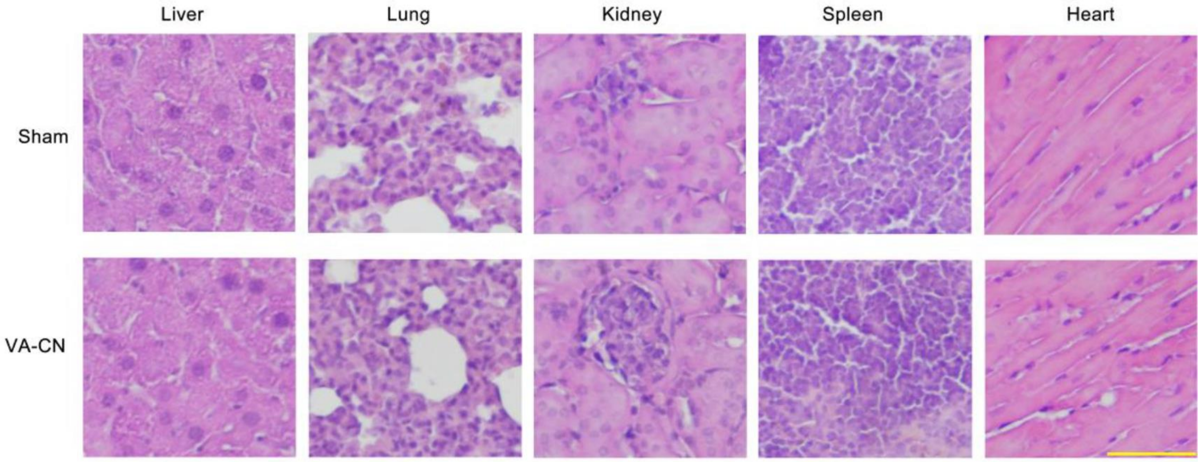

Supplementary Figure 2. In vivo toxicity analysis. Histological analysis of the liver, lung, spleen, kidney, and heart stained with hematoxylin and eosin in Sham and VA-CN treated rats at 4 weeks after injury (Scale bar: 50 µm).
